# Left Atrial Diameter is an Independent Predictor of Postoperative Atrial Fibrillation after Minimally Invasive Mitral Valve Surgery

**DOI:** 10.1186/1749-8090-10-S1-A25

**Published:** 2015-12-16

**Authors:** Andrew Cheng, Hansraj R Bookun, Michael Rasmussen, David Lee, Chin Siew Lee, Xiao Bo Zhang, Shivanand Gangahanumaiah, Marco Moscarelli, Giacomo Bianchi, Pier A Farneti, Marco Solinas

**Affiliations:** 1 Department of Cardiothoracic Surgery, University Hospital Geelong, Australia; 2 Department of Vascular Surgery, University Hospital Geelong; 3 Royal Australasian College of Surgeons, Melbourne, Australia; 4 Cardiology Research Unit, University Hospital Geelong, Australia; 5 Cardiothoracic Department, Fondazione Toscana G. Monasterio, Massa, Italy

## Background/Introduction

Postoperative atrial fibrillation (POAF) is the commonest arrhythmia after cardiac surgery, associated with longer hospital stay, increased morbidity and mortality. Development of POAF is certainly multifactorial, however the exact mechanism remains unknown. Left atrial (LA) enlargement is commonly associated with mitral valve disease.

## Aims/Objectives

Investigate the impact of LA diameter on the incidence of POAF in patients undergoing minimally invasive mitral valve surgery.

## Method

From September 2003 to December 2013, 1604 minimally invasive mitral valve replacement or repairs were performed. There were 577 patients with a prior history of atrial fibrillation who were excluded from analysis. POAF was identified by continuous cardiac monitoring.

Homogeneity of the sample was tested using multivariate regression, which did not identify any statistically significant confounding variables. Descriptive statistics were used to characterize samples with regards to demographic and perioperative variables. A logistic regression model was used to investigate the impact of LA diameter on the incidence POAF.

## Results

A total of 1027 patients were included in the analysis, of which 126 patients had concomitant tricuspid valve surgery and 85 patients underwent redo-surgery. There were 58% males (n = 595) and overall mean age was 59.6 ± 13.5 years. The overall mean LA diameter (mm) is 45.7 ± 7.7.

The incidence of POAF was 34% overall (n = 349). Females had a higher incidence of POAF compared to males (39% vs. 30%, p = 0.003). Mean age for patients with and without POAF was 60.2 ± 13.7 and 59.2 ± 13.4 respectively. Mean LA diameter was 47.2 ± 7.7 for patients with POAF and 44.9 ± 7.5 for patients without POAF (p < 0.001).

The logistic regression model parameters exhibited a beta-coefficient of 0.038 (p < 0.001; 95% CI: 0.021-0.055). For every millimeter increase in LA diameter, there is a corresponding increase in odds of POAF by a factor of 1.039 (p < 0.001).

## Discussion/Conclusion

In patients undergoing minimally mitral valve surgery we found LA diameter to be an independent predictor of POAF with a monotonic increase relationship.

